# Study on a job competence evaluation system for resident physicians (including integrated postgraduates) receiving standardized training

**DOI:** 10.1186/s12909-023-04833-w

**Published:** 2023-11-07

**Authors:** Yuanzheng Fu, Guoxiang Zhao, Jie Shan, Luxian Zeng

**Affiliations:** 1 Department of Science and Education, The Affiliated Guangdong Second Provincial General Hospital of Jinan University, Guangzhou, P.R. China; 2 Unions of Trade, The Affiliated Guangdong Second Provincial General Hospital of Jinan University, Guangzhou, P.R. China

**Keywords:** Job competence, Resident physician, Standardized training, Postgraduates pursuing a professional degree, Integrated education

## Abstract

**Background:**

Standardized training for resident physicians is the primary form of postgraduate medical education, and it plays a pivotal role in healthcare safety and industry stability. Currently, it has garnered significant attention from healthcare institutions.

**Methods:**

By conducting a comprehensive literature review and a Delphi consultation in June 2022 for which 40 experts in clinical medicine, public health, and other related fields in China were invited. The indicators were adjusted according to the results of the consultation, and the final indicator weights were determined through an analytic hierarchy process.

**Results:**

The response rate was 100%, and the expert authority coefficient was 0.879. The consistency among the experts on the tertiary indicators, as measured by Kendall’s *W*, was 0.675 (χ^2^ = 42.516, *p* < 0.001). Based on the results of the expert consultation, a job competence evaluation system for resident physicians (including integrated postgraduates) receiving standardized training was established, which included 6 primary indicators, 18 secondary indicators, and 116 tertiary indicators. The weights for the primary indicators, namely professional quality, skills and knowledge, patient care, communication and collaboration, teaching skills, and lifelong learning, were 0.313, 0.248, 0.181, 0.083, 0.066, and 0.110, respectively. The top three secondary indicators in terms of combined weights were clinical skills (0.122), professional ethics (0.120), and professional dedication (0.109). The three tertiary indicators with the highest scores were “maintains collaboration with peers and colleagues in patient treatment,” “has clinical thinking skills, makes diagnosis and treatment decisions based on analysis of evidence, and has the ability to administer suitable treatments,” and “abides by laws and discipline and refuses to seek personal gains in medical practice”; their combined weights were 0.035, 0.028, and 0.027, respectively.

**Conclusion:**

This study has established a concrete, objective, and quantifiable competency assessment index system for standardized training of clinical resident physicians (including integrated postgraduates). This system provides a foundation for the quantitative evaluation of the competency of clinical resident physicians (including integrated postgraduates) undergoing standardized training.

## Background

The standardized training of resident physicians is an essential part of postgraduate medical education; it is important because it (1) equips the physicians with the necessary medical knowledge and clinical skills to undertake diagnostic and treatment tasks in a standardized and independent manner, (2) improves the quality of medical care, and (3) safeguards public health [1]. In November 2014, the Chinese Ministry of Education, in collaboration with six other departments, including the National Health and Family Planning Commission and the State Administration of Traditional Chinese Medicine, issued the *Opinions on Deepening the Reform of Clinical Medical Talent Training through Medical-Educational Collaboration* (Education Research [2014] No. 2). This directive explicitly called for the standardization of resident physician training. Beginning in 2015, all newly enrolled clinical medical master’s degree graduate students, who also participate in standardized residency training, are required to undergo clinical training in accordance with the nationally established standardized requirements for resident physicians [2]. However, due to various factors, there remains no clearly defined integrated model for the convergence of standardized resident physician training and professional master’s degree graduate education. Furthermore, the lack of quantifiable assessment and evaluation criteria persists. Consequently, the development and mastery of a systematic training evaluation indicator system carries profound significance for advancing healthcare reform in China.

In 1973, renowned psychologist David McClelland at Harvard University introduced one of the earliest concepts of competency. He defined it as underlying characteristics that distinguish individuals with exceptional performance from their peers in a specific job. These characteristics may encompass motivation, personality traits, self-image, attitudes, values, domain-specific knowledge, cognitive or behavioral skills, and more. Notably, these traits significantly differentiate individuals with outstanding and average performance—a concept known as the “competency model” or “competency-based model“ [3]. In the 1980s, the concept of competency was applied to the study of clinical physicians. The goal was to outline the competencies required by qualified resident physicians. This approach, known as competency-based medical education, offers a detailed description of the competency requirements for resident physicians, enhancing the standardization of their training [4].

Some Western countries, following extensive research, have established national physician competency frameworks and competency-based education and training (CBET) models. Competency serves as the foundation for medical education, training, and assessments [5]. Subsequently, pertinent governmental bodies, institutions, and professional associations in developed countries embarked on extensive and in-depth studies of this educational model, seeking to establish national competency frameworks. For instance, in 1998, the Accreditation Council for Graduate Medical Education (ACGME) in the United States introduced a competency framework for clinical physicians, which included domains such as professionalism, patient care, medical knowledge, communication skills, practice-based learning, and system-based practice.This model functions as the foundational framework for postgraduate education and enjoys widespread implementation. To assess specialist physician competencies more effectively, the ACGME and the American Board of Medical Specialties (ABMS) developed the Toolbox of Assessment Methods based on their competency model [6]. This toolbox utilizes systematic and scientific evaluation methods to assess competency levels. In 2005, the Royal College of Physicians and Surgeons of Canada (RCPSC) introduced the CanMEDS Competency Framework, which categorizes the competencies required by specialist physicians into seven roles: medical expert, communicator, collaborator, manager, health advocate, scholar, and professional. Each role is detailed in terms of the fundamental knowledge, skills, and abilities indispensable for specialist physicians [7]. Furthermore, the RCPSC also developed the CanMEDS Assessment Tools Handbook to evaluate the competency levels of specialist physicians in each of these roles [8]. In 2010, the International Association for Medical Education published a report in *The Lancet* on the future of medical education in the 21st century. This report highlighted the third generation of medical education reform, which focuses on clinical competency as the foundation for medical education and talent development. This model has become a global trend in the development of medical education [9, 10].

The Chinese residency training system was initiated relatively late. Currently, the standardized training for resident physicians focuses on job competency. However, the assessment indicators for job competency are relatively generic compared to international standards. Therefore, how to further develop the content of competency is an important issue that needs to be addressed urgently [11]. In 2014, the *Notice of the National Health and Family Planning Commission on the Issuance of the Management Measures for Standardized Residency Training for Resident Physicians (Trial)* (National Health Science and Education Development [2014] No. 49) outlined the objective of standardized residency training for resident physicians: to nurture a high-caliber clinical physician workforce with a fundamental focus on cultivating job competency [12]. In 2018, a collaborative effort by the Chinese Elite Teaching Hospitals Alliance for Resident Physician Training yielded the *Alliance Consensus on Core Competencies for Resident Physicians*, aiming to establish a core competency framework aligned with the unique Chinese context. This consensus introduced six core competencies that resident physicians should possess: professionalism, knowledge and skills, patient care, communication and collaboration, teaching abilities, and lifelong learning [11]. Furthermore, this framework presented 3 to 4 sub-requirements for each competency indicator. However, when it comes to assessing job competency in clinical resident physicians (including integrated postgraduates), only general principles have been established, lacking specific third-level indicators and their corresponding weights. Therefore, there is a compelling need to refine the evaluation system for the job competency of resident physicians. This refinement will not only facilitate a more comprehensive appraisal of their job competency but also contribute to the standardization and enhancement of residency training practices. With this in mind, the objective of this paper is to use the Delphi expert consultation method to develop a set of assessment indicators for clinical resident physicians (including integrated postgraduates) [13–15]. In addition, we will employ the Analytic Hierarchy Process (AHP) to determine the relative weights of these indicators. The ultimate aim is to provide a solid foundation for the quantitative and optimized assessment of job competency in clinical resident physicians (including integrated postgraduates). This research holds theoretical significance and practical relevance for the advancement of the medical workforce, the elevation of healthcare standards, and the progress of medical education and healthcare reform.

## Objectives and methods

### Development of a draft version of the job competence evaluation indicators

This study undertook an extensive literature review spanning the years from 1990 to 2022 to investigate the job competency of clinical resident physicians. The research encompassed a wide array of domestic and international databases, including but not limited to Wanfang, CNKI, CQVIP, PubMed, Medline, and Web of Science. Our search employed a set of strategic keywords such as “Job competence,“ “Resident physician,“ “Standardized training,“ “Professional degree graduate,“ " Integrated education,“ and others. Initially, the literature screening process involved a rigorous assessment of abstracts to exclude irrelevant and duplicate publications, alongside merging similar sources. Concurrently, the selection of relevant indicators was meticulously conducted, paralleling a thorough analysis of physician competency literature in their respective roles. Furthermore, this study was significantly inspired by a variety of sources, which collectively contributed to the formulation of the initial draft of the evaluation indicator system framework. These sources included the Core Competency Framework for Physicians in the United States [16], Canada’s Competency Framework for Physicians [7], the Physician Competency Framework outlined in “Good Medical Practice” by the General Medical Council (GMC) of the United Kingdom in 2006 [17], the Competency Framework for Clinical Physicians developed by China Medical University [18], and the Core Competency Framework Consensus for Resident Physicians released by the Chinese Elite Teaching Hospitals Alliance for Resident Physician Training.

### Finalization of the job competence evaluation indicators based on expert consultation

#### Development of a questionnaire on expert consultation

A questionnaire was developed composed of the following parts: (1) an introduction to the background, purpose, and significance of the consultation, and a guide for filling out the questionnaire; (2) a survey gathering basic information about the respondents, such as their gender, age, specialties, years of work, title, and educational background; and (3) a form to collect the respondents’ expert opinions on the importance of each of the evaluation indicators for job competence. These opinions were collected through the use of 5-point Likert scales, where 1 = *not important at all*, 2 = *of little importance*, 3 = *of average importance*, 4 = *very important*, and 5 = *absolutely essential* [19]. The questionnaire also had an open field where the respondents could add additional comments and suggest changes to the evaluation indicators [20]. The indicators were then screened according to the following criteria: (1) mean importance score > 4.0, (2) coefficient of variation < 0.25, and (3) full-score ratio > 20% [21].

#### Determination of consulting experts

Experts were chosen for their familiarity with the characteristics of job competence, and were recruited using purposive sampling. To ensure geographical representation, experts were chosen from major tertiary grade A hospitals in Guangdong Province, and hospitals, universities, and research institutions in Hainan and Beijing. The selection criteria were as follows: (1) voluntary participation, (2) > 5 years of experience in the relevant fields, (3) with at least a bachelor’s degree and an intermediate or higher professional title, and (4) with comprehensive knowledge and understanding of the relevant issues. The final roster was composed of 40 experts.

#### Expert consultation

In June 2022, the questionnaires were distributed to the experts and completed through the online survey platform Wenjuanxing. The experts did not meet with each other and were unaware of each other’s responses. After collecting the answered questionnaires, the research group calculated the mean weight scores, coefficient of variation (CV), and full-score ratio of each indicator; they also used the expert opinions to decide whether to revise any indicators. In the first round of expert consultation in this study, the coefficient of concordance was 0.675 (χ^2^ = 2348.979, *P* < 0.001), with a variation coefficient ranging from 0.060 to 0.234. The experts unanimously agreed on the importance of the indicators, and their assessment opinions were well-coordinated. Therefore, there was no need for a second round of expert consultation [22, 23]. Furthermore, based on the criteria for indicator selection, the experts did not suggest any deletions or modifications to the evaluation indicator system. The overall number of evaluation indicator items and the framework remained unchanged. However, there were two suggested modifications to the wording of third-level indicator names. Specifically, for “Know the basic elements of the medical profession,“ experts recommended changing “Know” to “Know, be familiar with, and master.“ For “Reasonably accept criticism from patients, family members and colleagues,“ experts suggested changing “Rationally accept” to “Make self-adjustment and self-improvement based on.“ We have made corresponding modifications to each item as suggested, resulting in the final formation of the competency evaluation indicator system for standardized training of clinical resident physicians (including integrated postgraduates). This system comprises six primary indicators, eighteen secondary indicators, and one hundred and sixteen tertiary indicators.

#### AHP analysis for determining the weight of the indicators at all levels

AHP was formally introduced and developed by the American operations researcher Saaty in the mid-1970s. It provides a suitable analyzing method because AHP is a multi-criteria decision making technique that allows subjective as well as objective parameters to be considered in the decision making process. The hierarchical process provides the possibility of studying the problem as a whole, while paying attention to the interaction between intrahierarchical components. In this method, each decision making problem can be designed in a tree framework, so that the levels of the tree include the goals, criteria to achieve the goals, sub-criteria and finally, the understudy options. By breaking the problem into decision-making levels, it can focus on the smaller set of decisions [24, 25]. The AHP analysis was conducted as follows. (1) Establishing a Hierarchical Model: Drawing from Fariba Hassani’s research [25], a hierarchical model was formulated, comprising the overall goal level, the benchmark level, and the alternative level. The overall goal level represents the decision objectives, the benchmark level serves as the intermediary, and the alternative level signifies the candidate indicators. In this study, the competency evaluation indicator system for standardized training of clinical resident physicians (including integrated postgraduates) was categorized into three levels: the goal level for research purposes, the benchmark level for primary and secondary evaluation indicators, and the alternative level for tertiary evaluation indicators. (2) Constructing Judgment Matrices: Judgment matrices blend qualitative and quantitative analysis. Based on the 9-level ratio scale developed by Saaty (as outlined in Table 1), every indicator within each level was compared pairwise to generate the relative importance judgment matrix for that level [26]. The Saaty scale in this study was established by calculating the difference between the average importance scores assigned by experts to the indicators. Specifically, assuming Z_ij_ and Z_jk_ represent the average importance values for any two evaluation aspects, a judgment matrix is constructed. It is determined that if Z_ij_-Z_jk_ = 0, Z_ij_ and Z_jk_ are equally important, and the Saaty scale is set to 1. If 0.13 < Z_ij_-Z_jk_ < 0.26, Z_ij_ is slightly more important than Z_jk_, and the Saaty scale is set to 3. If 0.39 < Z_ij_-Z_jk_ < 0.52, Z_ij_ is considerably more important than Z_jk_, and the Saaty scale is set to 5. If 0.65 < Z_ij_-Z_jk_ < 0.78, Z_ij_ is significantly more important than Z_jk_, and the Saaty scale is set to 7. If Z_ij_-Z_jk_ > 0.91, Z_ij_ is extremely more important than Z_jk_, and the Saaty scale is set to 9. When the difference falls between the two scales, the Saaty scale is 2, 4, 6, or 8 [25]. Following these principles, two pairwise judgment matrices for primary indicators were created, as depicted in Table 2.(3)Consistency Check and Weight Calculation Results: Utilizing Yaahp 10.3 software, weights for each level and a consistency check were determined. After inputting the pairwise comparison scales for each matrix, a fundamental consistency check was performed. A fully consistent matrix has a consistency ratio of 0. A matrix is considered satisfactorily consistent when the consistency ratio is less than 0.1 [27]. If the consistency ratio exceeds 0.1, the matrix lacks consistency. Following the successful consistency check for all matrices, the Yaahp 10.3 software was employed to compute the weight results for each indicator element.

**Table 1:** Importance Scale Level 1–9 [28]

Importance Level	Scale Level
Indicator A and Indicator B are equally important	1
Indicator A is moderately more important than indicator B	3
Indicator A is strongly more important than indicator B	5
Indicator A is very strongly more important than indicator B	7
Indicator A is extremely more important than indicator B	9
The importance level of indicator A relative to indicator B falls between the descriptions mentioned above.	2, 4, 6, 8
Indicator B is slightly less important than indicator A	Take the reciprocal

Note: If the assigned value is less than 0, it means the level to which indicator A is less important than indicator B. If the reciprocal is taken, such as 1/3 for slightly less important and 1/9 for extremely less important. Similarly, the intermediate values for judgment are 1/2, 1/4, 1/6, and 1/8

**Table 2:** AHP Judgment Matrix for Primary Indicators

Mean importance		A_1_	A_2_	A_3_	A_4_	A_5_	A_6_
4.85	A_1_	1	2	2	3	3	3
4.83	A_2_	1/2	1	2	3	3	3
4.80	A_3_	1/2	1/2	1	3	3	2
4.65	A_4_	1/3	1/3	1/3	1	2	1/2
4.63	A_5_	1/3	1/3	1/3	0.5	1	1/2
4.68	A_6_	1/3	1/3	1/2	2	2	1

#### Calculation of relevant indicators and interpretation criteria

Relevant indicators were calculated as follows. (1) Enthusiasm coefficient of experts: Reflected by the rate of response to the questionnaire, meaning the number of experts who completed the questionnaire/the total number of invited experts × 100% [29].(2) The degree of expert authority: Based on the basis for the experts’ judgment (theoretical basis, practical experience, Peer opinions, and expert intuition) and familiarity with the issue (very familiar, moderately familiar, somewhat familiar, less familiar, and unfamiliar). The corresponding assigned scores are shown in Table 3. The authority coefficient (Cr) = (familiarity coefficient + judgment coefficient)/2, with Cr > 0.7 indicating a high level of expert authority [20]. (3) Full-score ratio of indicators = the number of experts giving full scores/the total number of invited experts × 100%.

**Table 3:** Items Used to Calculate Expert Authority Coefficients and Criteria for Assigning Values

Judgment Basis	Judgment coefficients			Familiarity	Familiarity coefficients
	Large	Moderate	Small	Very familiar	1
Theoretical basis	0.3	0.2	0.1	Moderately familiar	0.8
Practical experience	0.5	0.4	0.3	Somewhat familiar	0.5
Peer opinions	0.1	0.1	0.1	Less familiar	0.2
Expert intuition	0.1	0.1	0.1	Unfamiliar	0

### Statistical analysis

SPSS 20.0 was used for the statistical analysis, and yaahp 10.3 was used for the AHP analysis to calculate the weights of indicators at each level. Normally distributed measurement data were expressed as (x̄ ± s), whereas enumeration data were expressed in frequencies. Kendall’s *W* and χ^2^ testing were used to determine the degree of consistency of the expert opinions. AHP analysis was used to calculate the weights of indicators at each level. The combined weights of primary indicators were calculated based on the scores given by the experts, whereas the combined weight of the secondary indicator = the score of the secondary indicator × the combined weight of the primary indicator, and the combined weight of the tertiary indicator = the score of the tertiary indicator × the combined weight of the secondary indicator. A two-sided test with a testing level of α = 0.05 was conducted.

## Results

### Basic information of experts

The 40 enrolled experts were (42.13 ± 5.73) years old, and the number of years engaged in related work was (16.63 ± 7.30). Among them, 23 (57.5%) were male and 17 (42.5%) were female, 72.5% held a master’s degree or higher, and 77.5% held senior titles. The details are shown in Table 4.

**Table 4:** Basic Information of the Expert Panel (n = 40)

Characteristic		Count	Percentage
Gender			
	Male	23	57.5%
	Female	17	42.5%
Age	42.13 ± 5.73	40	
Specialties			
	Clinical/Technical	31	77.5%
	Administrative	4	10.0%
	Other	5	12.5%
Years of work	15.63 ± 7.30	31	
Professional title			
	Senior	12	30.0%
	Associate senior	19	47.5%
	Intermediate	8	20.0%
	None	1	2.5%
Academic qualification			
	Doctorate	14	35.0%
	Master’s	15	37.5%
	Bachelor’s	11	27.5%

### Expert enthusiasm and authority coefficients

In this study, the questionnaires were distributed to 40 experts, and all 40 questionnaires were recovered, with a valid response rate of 100%. The authority coefficient of the experts in this study was 0.879.

### Determination of Indicator weights by AHP analysis

#### Weights of primary and secondary indicators

The importance scores of the primary and secondary indicators were calculated based on the experts’ importance scores of the tertiary indicators. The judgment matrices constructed for the importance evaluation of the primary and secondary indicators both passed the consistency test and could be used to calculate the indicator weights. Professional quality, knowledge and skills, patient care, communication and collaboration, teaching skills, and lifelong learning were weighted as 0.313, 0.248, 0.181, 0.083, 0.066, and 0.110, respectively (see Table 5). The top three secondary indicators in terms of combined weights were clinical skills (0.122), professional ethics (0.120), and professional dedication (0.109).

#### Weights of tertiary indicators

For the tertiary indicators, the mean importance scores ranged from 4.38 to 4.925, and the CVs ranged from 0.05 to 0.21. The three tertiary indicators with the highest scores were “maintains collaboration with peers and colleagues in patient treatment,” “has clinical thinking skills, makes diagnosis and treatment decisions based on analysis of evidence, and has the ability to administer suitable treatments,” and “abides by laws and discipline and refuses to seek personal gains in medical practice,” and their combined weights were 0.035, 0.028, and 0.027, respectively.

**Table 5:** A System for Evaluating the Job Competence of Resident Physicians (and Postgraduates) Receiving Standardized Training

Primary/Secondary/Tertiary Indicator	x̄ ± s	CV	Combined Weight
Professional quality			0.313
Professional ethics			0.120
Maintains honesty and fairness in medical practice and stays away from profit-seeking activities	4.85 ± 0.36	0.07	0.016
Sticks to medical ethics and can properly handle ethical issues	4.83 ± 0.44	0.09	0.012
Has awareness of medical protection	4.73 ± 0.55	0.12	0.008
Has a rigorous and realistic working style	4.93 ± 0.26	0.05	0.026
Has compassion and respect for life	4.88 ± 0.33	0.07	0.021
Sticks to continuous self-improvement and pursues excellence	4.85 ± 0.36	0.07	0.017
Has good patience and endurance	4.70 ± 0.84	0.18	0.007
Maintains a good physician–patient relationship and sticks to honesty with patients	4.78 ± 0.42	0.09	0.010
Sticks to altruism and respects patients’ decisions	4.43 ± 0.95	0.21	0.003
Professional dedication			0.109
Holds professional values: technical excellence, dedication, responsibility, rigor, diligence, perseverance, etc.	4.85 ± 0.36	0.07	0.025
Has a passion for and confidence in work, and has a sense of vocation	4.70 ± 0.51	0.11	0.008
Is able to solve problems, manage time, and keep improving the work	4.73 ± 0.50	0.11	0.013
Informs the patient and their family members and seeks their consent first before examination or treatment	4.78 ± 0.47	0.10	0.019
know, be familiar with, and master of the basic elements of the medical profession	4.73 ± 0.45	0.09	0.015
Abides by the law, is disciplined, and refuses to seek personal gains in the practice of medicine	4.85 ± 0.36	0.07	0.027
Humanistic literacy			0.040
Respects patients’ gender and cultural diversity, beliefs, and autonomy	4.70 ± 0.56	0.12	0.006
Accepts constructive and appropriate criticism	4.60 ± 0.58	0.13	0.003
Has the abilities of self-monitoring and psychological adjustment, can seek help when necessary	4.58 ± 0.67	0.15	0.003
Listens to patients‘ family members	4.55 ± 0.59	0.13	0.002
Has confidence	4.68 ± 0.57	0.12	0.005
Has rational self-understanding and judgment and can proactively learn and undertake self-improvement	4.73 ± 0.50	0.11	0.007
Has the abilities of self-regulation and stress tolerance	4.75 ± 0.43	0.09	0.009
Has mastery of the medical-related social sciences, such as the history of medicine, philosophy of medicine, and medical ethics	4.63 ± 0.62	0.13	0.004
Provides palliative care to alleviate symptoms	4.45 ± 0.84	0.19	0.001
System improvement			0.044
Has mastery of the knowledge knowledge of public health	4.45 ± 0.71	0.16	0.002
Has mastery of the basic knowledge of health care laws	4.53 ± 0.63	0.14	0.002
Has mastery of the knowledge of social science and medical ethics	4.55 ± 0.71	0.16	0.003
Has mastery of the basic knowledge of applied clinical research and teaching	4.58 ± 0.63	0.14	0.003
Has the ability to take histories from patients accurately and completely	4.78 ± 0.47	0.10	0.010
Has the ability to select appropriate ancillary examination methods and analyze the results	4.75 ± 0.49	0.10	0.008
Has the ability to report and analyze clinical issues	4.73 ± 0.50	0.11	0.007
Has the ability to identify medical risks and ensure medical safety	4.75 ± 0.49	0.10	0.006
Has the ability to identify and report infectious diseases in time in accordance with regulations, and prevent and control common infectious diseases	4.68 ± 0.61	0.13	0.005
Knowledge and skills			0.248
Theoretical knowledge			0.048
Masters knowledge and skills in applied clinical medicine	4.83 ± 0.38	0.08	0.023
Masters basic knowledge of biology and medicine	4.55 ± 0.67	0.15	0.007
Has mastery of computer literacy (e.g., the use of recording systems, software, and scientific tools)	4.43 ± 0.77	0.17	0.004
Has the ability to read English literature and masters basic English speaking and writing skills	4.38 ± 0.73	0.17	0.003
Has the motivation to keep learning with a modest attitude, and participate in medical training and continuing education	4.65 ± 0.57	0.12	0.011
Clinical skills			0.122
Has the ability to take histories from patients	4.80 ± 0.40	0.08	0.021
Has the ability to examine physical conditions and mental status of patients	4.78 ± 0.42	0.09	0.016
Knows the diagnostic process and has the ability to analyze and interpret diagnostic results	4.78 ± 0.42	0.09	0.016
Has the ability to identify critical conditions and provide first aid or other emergency treatments	4.85 ± 0.42	0.09	0.025
Has the ability to properly use medical equipment and health care facilities	4.65 ± 0.57	0.12	0.008
Has the ability to record observation results and give verbal or written instructions in an accurate and timely manner	4.73 ± 0.45	0.09	0.011
Has the ability to administer drugs safely and manage drugs effectively	4.68 ± 0.52	0.11	0.010
Has the ability to issue legitimate medical certificates (e.g., for insurance or funeral-related purposes)	4.63 ± 0.53	0.12	0.006
Focuses on patient safety and quality of care	4.63 ± 0.62	0.13	0.006
Has the ability to write standard medical records and has the habit of keeping and analyzing such records for improvement	4.60 ± 0.89	0.19	0.005
Clinical thinking			0.077
Possesses a clinical thinking mindset, is able to make diagnosis and treatment decisions based on the analysis of evidence, and administer suitable treatments	4.80 ± 0.40	0.08	0.028
Has the ability to select appropriate diagnostic and therapeutic options based on the principles of evidence-based medicine	4.55 ± 0.95	0.21	0.006
Knows when and how to seek help from supervising physicians	4.73 ± 0.50	0.11	0.017
Focuses on patient safety and quality of care	4.73 ± 0.59	0.13	0.016
Has the ability to identify, manage, or mitigate risks (e.g., suicide)	4.60 ± 0.62	0.14	0.010
Patient care			0.181
Patient management			0.056
Has the expertise to address patients’ physical symptoms	4.80 ± 0.40	0.08	0.017
Has the ability to identify patient discomforts and administer relevant treatments	4.70 ± 0.51	0.11	0.012
Has the ability to mitigate patient anxiety and concern	4.60 ± 0.58	0.13	0.007
Has the ability to provide emotional support to patients	4.55 ± 0.67	0.15	0.005
Has the ability to sympathize with patients	4.55 ± 0.63	0.14	0.005
Has the ability to meet patients’ needs and encourage them	4.48 ± 0.84	0.19	0.003
Protects patients’ right to information and privacy	4.60 ± 0.58	0.13	0.007
Patient education			0.035
Has the ability to describe in detail patients’ conditions and relevant treatment plans	4.63 ± 0.53	0.12	0.019
Allows patients and their family members to choose treatment plans as appropriate	4.48 ± 0.84	0.19	0.007
Has the ability to use technical means to educate patients on the treatment of their conditions	4.55 ± 0.63	0.14	0.009
Clinical decision making			0.089
Maintains collaboration with peers and colleagues in patient treatment	4.75 ± 0.43	0.09	0.035
Assesses patients’ physical conditions and medical histories when developing treatment plans	4.68 ± 0.57	0.12	0.025
Does their best to alleviate patient discomforts	4.63 ± 0.53	0.12	0.015
Makes diagnosis and treatment decisions together with patients and their family members and relevant specialists	4.63 ± 0.62	0.13	0.015
Communication and collaboration			0.083
Physician-patient communication			0.042
Has the ability to effectively convey messages to patients and their family members and colleagues	4.65 ± 0.48	0.10	0.004
Understands, trusts, and supports patients and their family members	4.65 ± 0.57	0.12	0.004
Masters non-verbal communication skills, such as appropriate professional image and body language and expressions	4.70 ± 0.56	0.12	0.007
Has the ability to resolve physician–patient conflicts and establish good physician–patient relationships	4.73 ± 0.45	0.09	0.010
Has the ability to communicate negative information to patients and their family members in a timely and tactful manner	4.70 ± 0.51	0.11	0.007
Has the ability to calm patients’ anger or dispel misunderstandings	4.73 ± 0.45	0.09	0.010
Teamwork			0.021
Manages time well	4.70 ± 0.46	0.10	0.005
Understands the responsibilities of team members and maintains close collaboration with them	4.70 ± 0.51	0.11	0.005
Has the ability to resolve conflicts between team members	4.58 ± 0.54	0.12	0.002
Participates in interdisciplinary and interdepartmental teamwork	4.63 ± 0.53	0.12	0.003
Likes to help colleagues and other health care professionals	4.65 ± 0.53	0.11	0.003
Makes continuous assessment and helps improve team performance	4.58 ± 0.59	0.13	0.002
Leadership			0.021
Can adapt to complex and uncertain events	4.63 ± 0.66	0.14	0.003
Keeps improving leadership and management skills in practice	4.55 ± 0.63	0.14	0.002
Arranges workload in a reasonable way and undertakes career planning	4.73 ± 0.50	0.11	0.005
Knows how to present and report work content and results	4.65 ± 0.57	0.12	0.007
Proactively learns the areas of expertise and specialties of each department and health care professional for the sake of providing better clinical services and making better clinical decisions	4.63 ± 0.58	0.13	0.003
Teaching skills			0.066
Clinical teaching			0.049
Has the ability to lecture on theories in the professional area	4.63 ± 0.53	0.12	0.008
Tutors interns	4.55 ± 0.67	0.15	0.003
Encourages interns and provides them with hands-on experience	4.55 ± 0.63	0.14	0.003
Has the ability to identify problems through clinical practice and summarize professional knowledge and theories	4.63 ± 0.53	0.12	0.008
Has the communication and expression skills for on-site teaching	4.70 ± 0.51	0.11	0.010
Helps students consolidate the taught knowledge	4.60 ± 0.62	0.14	0.005
Values discussion, interaction, and timely feedback in teaching	4.60 ± 0.62	0.14	0.005
Has the ability to develop innovative teaching methods and use modern multimedia information technology to facilitate teaching	4.58 ± 0.63	0.14	0.004
Has the ability to compile teaching resources such as audio recordings, short videos, and clinical cases	4.48 ± 0.71	0.16	0.002
Medical science popularization			0.016
Recognizes the value of science popularization and raises the awareness of science popularization	4.48 ± 0.59	0.13	0.005
Has a solid grasp of medical theories and knowledge for in-depth learning	4.48 ± 0.63	0.14	0.005
Has the ability to develop theories and present them in writing	4.45 ± 0.63	0.14	0.003
Has good literacy for science popularization and can identify helpful science content	4.40 ± 0.70	0.16	0.002
Has the ability to popularize science content using new media and videos	4.40 ± 0.73	0.17	0.002
Lifelong learning			0.110
Evidence-based medicine and critical thinking			0.029
Continuously updates professional knowledge and strives to improve professional skills in clinical practice	4.68 ± 0.57	0.12	0.008
Does well in finding out their shortcomings and limitations in clinical practice	4.60 ± 0.62	0.14	0.004
Make self-adjustment and self-improvement based on criticism from patients and their family members and colleagues	4.58 ± 0.63	0.14	0.005
Optimizes the knowledge structure through continuous learning of information technology, data analysis, literature research, etc. related to clinical medicine	4.53 ± 0.63	0.14	0.002
Adheres to the principles of evidence-based medicine and is good at verifying theories against clinical evidence to improve the effectiveness of clinical practice	4.55 ± 0.71	0.16	0.003
Has good critical thinking skills in clinical practice	4.63 ± 0.62	0.13	0.006
Self-improvement			0.045
Understands their own strengths and weaknesses and takes the initiative to undertakes self-improvement	4.65 ± 0.53	0.11	0.006
Has objective judgment about their own expertise and can identify problems in time	4.58 ± 0.54	0.12	0.012
Participates in continuing education and in-service training	4.58 ± 0.63	0.14	0.006
Puts information from various sources into perspective	4.65 ± 0.53	0.11	0.009
Keeps improving their abilities to learn medical-related legal knowledge and enhance physician-patient communication	4.63 ± 0.53	0.12	0.013
Academic research			0.036
Takes the initiative to prepare and publish academic papers, study reports, etc.	4.60 ± 0.49	0.11	0.003
Masters and uses basic research methods	4.60 ± 0.54	0.12	0.003
Bases academic research on clinical issues	4.65 ± 0.57	0.12	0.004
Thinks creatively and critically	4.68 ± 0.52	0.11	0.009
Collects data through literature analysis, statistics, and other research tools	4.58 ± 0.54	0.12	0.005
Participates in research activities in the field of health care	4.58 ± 0.54	0.12	0.007
Effectively coordinates research institutions, leaders, and other relevant resources	4.55 ± 0.59	0.13	0.005

## Discussion

In the context of the ongoing global medical education reform, the primary focus has shifted towards job competency, with a specific emphasis on elevating the job competency of clinical physicians [30]. This study employs a combination of the Analytic Hierarchy Process (AHP) and the Delphi method to construct an evaluation indicator system for the competency of clinical resident physicians (including integrated postgraduates). Both of these methods are based on the expert group’s theoretical knowledge and practical experience [31, 32]. The AHP, as a method for decision-making, incorporates mathematical and statistical processing on top of expert subjective judgments, enhancing its logical foundation and making it more scientifically rigorous [33]. Moreover, through the inherent logic at each hierarchical level, different indicators are assigned appropriate weights, allowing the evaluation system to be focused while comprehensively reflecting the core competencies that clinical resident physicians (including integrated postgraduates) should possess from various perspectives.Compared to the purely qualitative methods used in the past, this research method results in a more objective and scientific evaluation indicator system.

The competency evaluation system constructed in this study comprises six primary indicators, eighteen secondary indicators, and one hundred and sixteen tertiary indicators. It quantifies competency in six crucial dimensions: professional qualities, knowledge and skills, patient care, communication and collaboration, teaching abilities, and lifelong learning. This quantification enhances comparability and objectivity. Furthermore, the establishment of weight coefficients for primary and secondary indicators enhances the practical applicability of these indicators in real-world scenarios. It not only provides an objective foundation for quantitatively assessing the competency of clinical resident physicians undergoing standardized training (including integrated postgraduates) but also offers a clear and intuitive competency development framework for training institutions. This framework can act as a tangible target in future training programs and clinical teaching, ultimately leading to more effective training outcomes.

In this study, an indicator-based job competence evaluation system for resident physicians (and postgraduates) receiving standardized training was preliminarily developed based on literature analysis and expert discussion. Professional titles, specialties, educational background, job positions, and years of work were used as the criteria for including consulting experts, and a total of 40 experts were ultimately enrolled [34]. Among the 40 experts, the average number of years engaged in related work was 16.50 ± 7.30, showing extensive work experience; 72.5% held a master’s degree or higher, and 77.5% held senior titles. The cohort of enrolled experts could thus be considered highly qualified. The valid response rate of the expert consultation was 100%, and the authority coefficient was 0.879 (> 0.7), indicating that the experts were highly motivated to provide advice, and the consultation results were authoritative and reliable. The Kendall‘s *W* of the expert responses was 0.675 (χ^2^ = 42.516, *p* < 0.001), indicating that the expert opinions were highly consistent. The AHP was introduced to test the consistency of the indicators at all levels. The result *Cr* < 0.100 suggests that the indicators were unambiguous without logical errors [27].

Based on the AHP analysis, the weights of the primary indicators, namely professional quality, knowledge and skills, patient care, communication and collaboration, teaching skills, and lifelong learning, were 0.313, 0.248, 0.181, 0.083, 0.066, and 0.110, respectively. As demonstrated, professional quality had the highest weight, which is consistent with the findings of the study by Liao Wumeizi [35]. Professional quality refers to the self-imposed moral restraint of physicians and is the soul of their medical career. The *Content and Standards of Standardized Resident Training (2020 Revised Edition)* suggests that the goal of resident training for medical institutions at all levels is to foster resident clinicians with good professional quality and expertise who are ready to provide humanitarian medical service to patients and who can independently diagnose and treat common diseases within their specialty [36]. The indicator “knowledge and skills” encompasses knowledge and skills in basic medicine, clinical medicine, and related disciplines essential for resident physicians. These two indicators complement each other and are both indispensable. As the paradigm of medical care changes and physician–patient conflict becomes more acute, patient care and communication and collaboration also become increasingly important, which explains why their weights are also high. Finally, teaching skills and lifelong learning are paths for self-improvement in the career of physicians.

The top three secondary indicators were clinical skills (0.122), professional ethics (0.120), and professional dedication (0.109). Clinical skills serve as the cornerstone of a medical professional’s core competencies. A strong command of clinical skills is an indispensable prerequisite for individuals seeking to embark on the path of standardized training as clinical resident physicians, including integrated postgraduates. Notably, clinical skills carry the heaviest weight within the competency evaluation system. This weightage mirrors the findings of Liu Zhuang and fellow researchers in their endeavors to construct a comprehensive competency indicator system for clinical physicians [37].

The three tertiary indicators with the highest scores were “maintains collaboration with peers and colleagues in patient treatment,” “has clinical thinking skills, makes diagnosis and treatment decisions based on analysis of evidence, and has the ability to administer suitable treatments,” and “abides by laws and discipline and refuses to seek personal gains in medical practice”; their combined weights were 0.035, 0.028, and 0.027, respectively. These results show it is important for physicians to be able to assimilate into the team, know their responsibilities, help each other, avoid conflicts, effectively implement medical decisions, and abide by the law in the practice of medicine. In the daily responsibilities of clinical physicians, consistent communication and engagement with patients, their families, higher-level hospitals or specialty facilities, and colleagues are indispensable. Having robust interpersonal communication and teamwork skills serves as the fundamental requirement for fulfilling their roles. The Accreditation Council for Graduate Medical Education has identified interpersonal skills and communication abilities as one of the six essential competencies in the training of family medicine residents in the United States. This requires family medicine residents to effectively communicate and collaborate with patients, their families, and other healthcare professionals [38]. The results also show that it is important for clinical residents (and postgraduates) to develop clinical thinking as part of the diagnosis and treatment process [39].

By comparing our findings with those of other studies conducted domestically and internationally, we have found that, in terms of first-level indicators, there is a fundamental similarity in content, although there may be some differences in the way they are expressed. For example, in Canada, the competency indicator system’s first-level indicators are categorized into seven roles for general practitioners, such as communicators, managers, researchers, etc [7]. In contrast, other countries, including our indicator system, treat various fundamental abilities as first-level indicators. The clinical physician competency framework proposed by the Accreditation Council for Graduate Medical Education in the United States includes professional ethics, patient care, medical knowledge, communication skills, practice-based learning, and system-based practice [16]. Building upon these foundations, our study further refines second and third-level indicators, enriching the competency assessment system. This refinement provides an objective basis for quantifying and optimizing the evaluation of standardized training for clinical resident physicians (including integrated postgraduates).

As the Delphi method is based on experts’ subjective judgment, this study may have some limitations in terms of the indicators that were screened out of the study. In addition, the indicator system for evaluating the job competence of clinical resident physicians (and postgraduates) in standardized training that was established in this study has not yet been piloted in relevant institutions, therefore its reliability and validity need to be further verified in practice.

In summary, the indicator system for job competence evaluation of resident physicians (and postgraduates) receiving standardized training that has been preliminarily established in this study has been proven to have scientific and application value, and it can be a basis for quantitatively assessing the job competence of trainees. It can also serve as a reference for developing curriculum systems, rotation plans, and assessment criteria for postgraduates pursuing a master’s degree in clinical medicine.

## Data Availability

All data generated or analyzed during the current study are available from the corresponding author upon reasonable request.
